# Towards a collaborative interdisciplinary systems approach to urban food system transformation: a case study from the Mandala research consortium

**DOI:** 10.1098/rstb.2024.0155

**Published:** 2025-09-18

**Authors:** Alexia Sawyer, Kelly Parsons, Jean Adams, Soujanya Mantravadi, Steven Cummins, Martin White

**Affiliations:** ^1^ MRC Epidemiology Unit, School of Clinical Medicine, University of Cambridge, Cambridge CB2 0SL, UK; ^2^ Department of Engineering, University of Cambridge, Cambridge CB2 1PZ, UK; ^3^ Population Health Innovation Lab, Department of Public Health, Environments & Society, London School of Hygiene & Tropical Medicine, London WC1E 7HT, UK

**Keywords:** food systems, interdisciplinary science, stakeholder collaboration, systems science, transformation, diet

## Abstract

Despite the need to transform food systems to improve human and planetary health, the activities and actors of food systems remain largely intransigent to change. The Mandala research consortium offers a case study of how three methodological principles—interdisciplinary science, systems thinking and stakeholder collaboration—can be integrated to identify interventions with transformative potential in an urban food system. An existing conceptual framework was employed to interrogate the case study and reflect on the challenges and opportunities of its methods across two phases: (i) understanding the food system and identifying places to intervene; (ii) envisioning and prioritizing food system interventions. Ambitions to work across the breadth of the urban food system were supported by interdisciplinary science and stakeholder collaboration; tailoring the research process accommodated the epistemic, social, symbolic, spatial and temporal differences between stakeholders. A complex adaptive systems approach enabled identification of promising food system interventions. Nevertheless, challenges arose in every aspect of the work. A collaborative interdisciplinary systems approach to system transformation was articulated using a real-world example. While opportunities support the funding and design of research using a similar methodological approach, the documented challenges of adopting this approach may be overcome with practical solutions.

This article is part of the theme issue ‘Transforming terrestrial food systems for human and planetary health’.

## 1. Background

Transformation of food systems is urgently needed to reduce their substantial external costs to human and planetary health [1–3]. These systems are complex, comprising numerous, diverse actors and activities, which lead to emergent health, environmental and economic outcomes that are highly heterogeneous—from obesity to greenhouse gas emissions and commercial profits [2]. Modern food systems, particularly in high-income countries, are dominated by large commercial companies that operate alongside small and medium-sized companies, social enterprises and public sector organizations providing food for human consumption [4]. At every scale—from global to local, actors and activities interact dynamically in these systems to influence the stocks and flows of food, materials, human resources, information, money and intangible properties such as cultural beliefs and power. Thus, food systems can be characterized as complex adaptive systems [5,6].

A generalizable characteristic of complex adaptive systems is self-organization, which allows a system to restore a level of (spontaneous) order following disruption, so that it continues to achieve its embedded goals [7,8]. This reversion to order means there is in-built resistance to long-term system change [7,8]. Consequently, there is debate about how system transformation might be achieved.

Mandala (https://www.mandala-consortium.org/) is a consortium delivering a five-year research programme funded by the UK Research & Innovation’s Transforming UK Food Systems programme. Mandala aims to catalyse and evaluate change in the food system of the city of Birmingham, UK, with the objective of improving human and planetary health while reducing health inequalities and maintaining economic viability for food system actors. Birmingham has a sizeable urban food system, serving a culturally and socioeconomically diverse population of >1.1 million residents [9]. In the 2023 Food System Strategy, Birmingham City Council declared a need to ‘create a fair, sustainable and prosperous food system and economy’ [10, p. 2]. The city of Birmingham offers a setting ripe for food system change.

The complex, systemic nature of Mandala’s research challenge—spanning multiple activities, outcomes and processes—necessitates collaboration between a range of scientists and between those scientists and local food system actors. Research of this nature, aimed at solving complex, cross-disciplinary problems, is increasingly the focus of funding in the field of food systems, but operationalization of the challenges and opportunities of such research is hindered by a lack of documentation of research implementation.

### (a) A framework for interdisciplinary collaboration

Existing literature establishes a need for partnership between scientific disciplines and with food system stakeholders, as a way to bring together a plurality of knowledge to build a systems understanding, address complexity and uncertainty, and pursue actionable solutions [11,12]. However, despite consensus on interdisciplinary collaboration as a useful goal, the means to deliver it effectively are less well established: the term 'collaboration' is applied loosely, in a variety of ways, and rigorous appraisal of its application and effectiveness is often overlooked [11,13].

While no comprehensive theory of scientific collaboration exists [14], a range of frameworks have been proposed to identify and assess the dimensions of collaborative research. For example, Boix Mansilla *et al.* distinguish the cognitive–emotional–interactional aspects of successful interdisciplinary collaborations [15]. Moreover, Wiek *et al.* evaluate effectiveness according to a project’s problem focus, transformational research methodology, stakeholder participation, actionable results and larger impacts [16].

In their examination of the ‘conditions’ for collaboration, Freeth & Caniglia consider how epistemic, social, symbolic, spatial and temporal dimensions shape collaboration processes [13]. They note that the challenges of collaboration often lead to project designs and claims of ‘integrative interdisciplinarity’ (different disciplines working together to achieve shared understanding and learning) being reduced to ‘additive multidisciplinarity’ (different disciplines each contributing in isolation but with open channels of communication).

In this paper, we add to the corpus of ‘science on team science’, by reflecting on Mandala’s adoption of three methodological principles: interdisciplinary science, systems thinking and stakeholder collaboration, to identify potentially transformative interventions in an urban food system [14]. Our aims are twofold: first, to present a rich description of a real-world example combining interdisciplinary science, systems thinking and stakeholder collaboration by documenting the first two phases of Mandala, and second, to use Freeth & Caniglia’s framework of interdisciplinarity [13] to identify the challenges and opportunities of combining these methodological principles in an effort to move towards a collaborative interdisciplinary systems approach. Given Mandala research is ongoing, this paper does not attempt to evaluate Mandala’s success in understanding the food system or identifying interventions with the potential to catalyse food system change, which will be the subject of future publications.

## 2. Methods

### (a) Case study of a collaborative interdisciplinary systems approach

The Mandala research process was documented through analysis of project documents, discussion with the research team and the authors’ deep knowledge of the process. Worked examples were drawn from across Mandala to illustrate methods or processes.

### (b) Application of a conceptual framework

Where deemed relevant, Freeth & Caniglia’s five dimensions provided a framework against which to organize and analyse our critical reflections on the challenges and opportunities of applying Mandala’s three methodological principles (table 1) [13]. While Freeth & Caniglia focus on researcher–researcher collaboration, we extended their framework to encompass the researcher–stakeholder collaboration that characterizes Mandala. While critical reflections were initially generated in discussions between A.S. and K.P., they were later reviewed and supplemented by all authors.

**Table 1.** Five dimensions shaping collaborative research [13].

dimension	definition
epistemic	different assumptions about which research questions are central, how knowledge should be produced and what constitutes good knowledge
social	different ways of being together in research; relations with both peers and competitors; emotional dynamics of interdisciplinary collaboration
symbolic	power differentials, and how these differentials manifest in implicit and explicit ways; how power dynamics shape values, norms and expectations
spatial	ways in which different spaces enable or constrain collective research work; sense of belonging within a research community
temporal	different (expectations around) tempos, time regimes and forms of time

### (c) Case study of a collaborative interdisciplinary systems approach: the Mandala research process

The phases of Mandala are as follows (work packages displayed in electronic supplementary material, file S1): (i) identifying stakeholders and mapping the urban food system (work package 1) (ii); identifying and prioritizing interventions for delivery in the urban food system (work package 3); and (iii) delivery and evaluation of interventions and assessment of system change (work packages 4−6). The first two phases are outlined and reflected upon in this paper. The third phase is underway at the time of writing. As this paper is concerned with the research process, further specific outputs of the first two phases, such as causal loop diagrams (CLDs) representing parts of the food system, will be reported separately.

**Figure 1.**
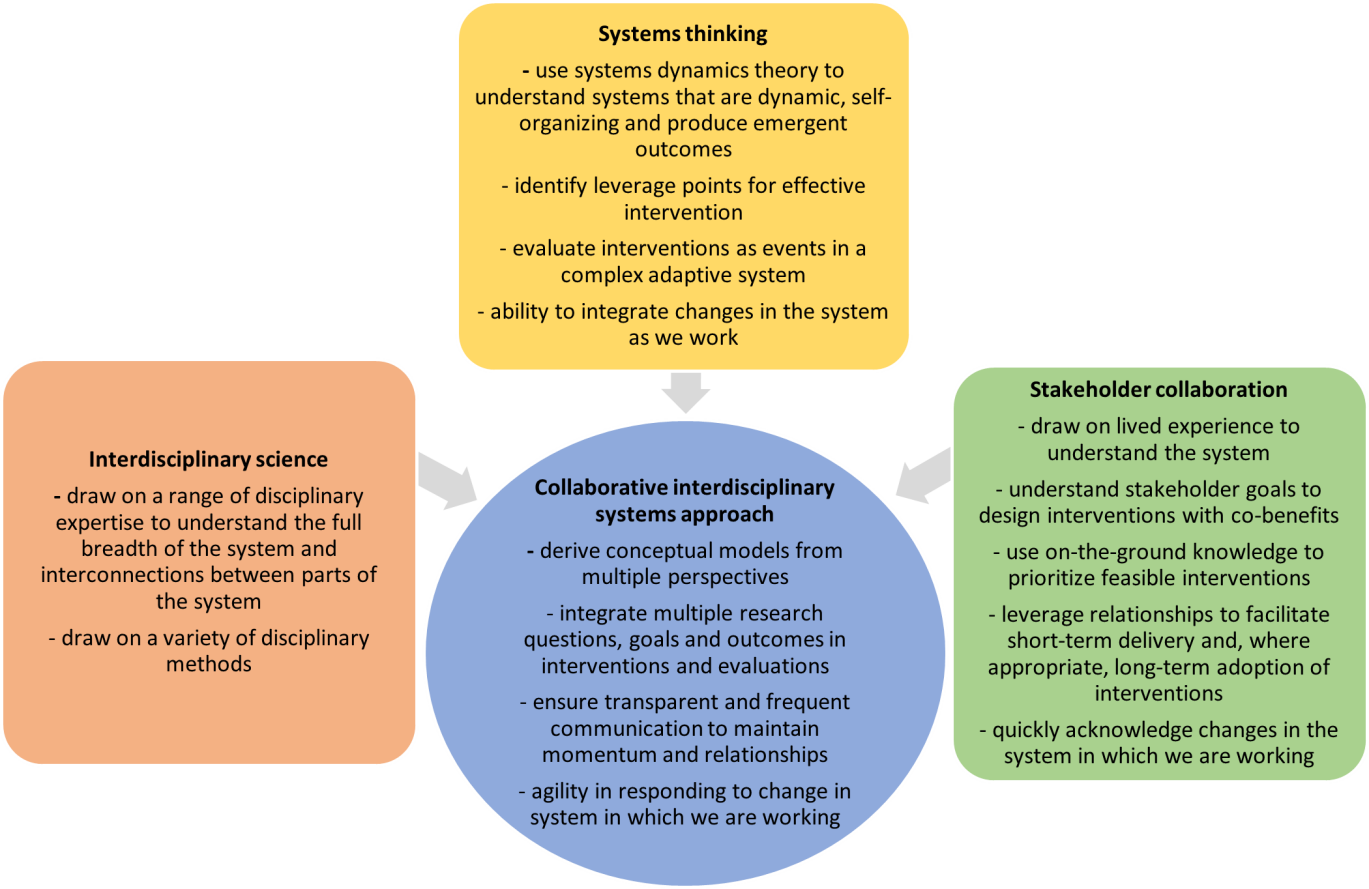
Visualization of perceived commonalities and unique contributions of the three methodological principles adopted in Mandala.

Three complementary methodological principles were embedded in the proposed design of Mandala to enable fuller acknowledgement and negotiation of the complexity of the urban food system to achieve system-level change: interdisciplinary science, systems thinking and stakeholder collaboration [17]. These three principles are operationalized in the context of Mandala in figure 1. In figure 1, we also articulate the overarching aspiration to apply them synergistically to enable a ‘collaborative interdisciplinary systems approach’.

Adopting an interdisciplinary scientific approach helped us to assess the whole breadth of the urban food system, by providing a range of analytical lenses and a comprehensive methodological arsenal for understanding the system and evaluating change [14]. The following disciplines were represented in the research team: public health (epidemiology and evaluation), social sciences (geography, sociology, psychology and behavioural sciences, policy studies, macro- and health-economics), system sciences, environmental and ecological sciences, mathematical modelling, business studies, engineering and manufacturing (supply chain analysis) and stakeholder engagement.

The project also adopted a complex adaptive systems approach. Rooted in system dynamics theory, a systems approach can be used to: (i) describe the (perceived) mechanisms driving emergent outcomes; (ii) identify places in the system with the greatest potential leverage for sustainable change; and (iii) design and evaluate interventions as events in the system, to understand their potential impacts on system behaviour and outcomes [18].

Lastly, Mandala aimed to collaborate with food system stakeholders at every stage of the research process. Close collaboration enables a shared understanding of how the urban food system leads to certain outcomes, encourages shared decision-making during intervention development to ensure that proposed system interventions are acceptable and feasible, and facilitates authentic consultation processes to enable rigorous, real-world evaluation. Thus, collaboration between academic and social stakeholders can be a cornerstone of transformative knowledge generation and solutions [19–21].

With the integration of these methodological principles, Mandala’s research process developed iteratively over time, through discussions between disciplines and with diverse stakeholder groups, informed by emergent learning about the complexities of the urban food system. Methods that were deemed possible in theory were not always possible or desirable in practice. Next, we detail the methods employed in the first two phases of Mandala.

#### (i) Phase 1a: defining sub-system boundaries and identifying relevant stakeholders

Bounding the system into sub-systems delineated what was within and outside our definition of an urban food system and ensured that subsequent mapping of the system was manageable and informed by representative stakeholder groups. A rapid, non-systematic, scoping review of diagrammatic visualizations of the food system in the published and grey literature was used to distil multiple characterizations of the food system into component parts comprising actors and/or activities. A table of these food system components was discussed within the Mandala research team and components were categorized into five sub-systems, to reflect core urban food system activities and relevant research team knowledge and expertise: supply chains and wholesaling (including: wholesale markets); grocery retail (including: supermarkets, convenience stores); institutional catering (including: schools, hospitals, prisons and workplaces); out-of-home retailing (including: restaurants, cafes, takeaways) and community food and support services (including: food banks, community food-growing projects, out-of-term children’s food programmes). Activities concerned with food waste use and disposal were considered within each of the five sub-systems. A team of Mandala researchers was convened to work on each of the sub-systems, with representation from relevant disciplines.

The types of actors involved in the sub-systems were listed in a stakeholder table, drawing on the rapid scoping review and team discussion. Specific individuals or organizations working in Birmingham’s food system were matched to actor types, with input from Birmingham City Council’s Food System Team. Forty-two stakeholders were invited to join the ‘Mandala Food System Cohort’—a longitudinal, qualitative study of urban food system actors—and participate in an initial interview on their role in the food system and food system change in Birmingham.

#### (ii) Phase 1b: articulating the problem: system outcomes of interest

We held an internal workshop of the Mandala research team to build consensus on the most important outcomes regarding health, environmental sustainability, equity and economic viability relevant to each food sub-system. Although we aimed to detail specific outcomes, this was deemed not possible without knowledge of which interventions would be evaluated within Mandala. Guided by the EAT–*Lancet* Commission on Healthy Diets from Sustainable Food Systems [22], outcomes were therefore characterized as a ‘direction of travel’ towards (i) increased consumption of fruit, vegetables and wholefoods and reduced consumption of red and processed meats and ultra-processed foods (hereafter referred to as ‘a healthy and sustainable diet’) and (ii) a food system that promoted these consumption patterns through equitable availability and affordability of relevant foods and reduced food waste across the value chain. Further specification of outcomes was postponed, allowing us to generate outcomes specific to each evaluation.

#### (iii) Phase 1c: mapping the sub-systems: developing causal loop diagrams

For each sub-system, causal loop diagramming (CLD, a visual depiction of hypothesized or assumed causal relationships between variables in a system) was used to map the mechanisms underpinning low levels of provision or consumption of healthy and sustainable diets. Following internal piloting and to reduce demands on stakeholders, an initial iteration of each causal loop diagram (CLD) was developed using insights from our initial food system stakeholder interviews, existing evidence detailing the factors and relationships pertinent to the sub-system, and sub-system team knowledge. For the out-of-home retailing sub-system, a recent CLD developed using group model building with relevant stakeholders was adapted and supplemented using team knowledge [23]. To ensure a consistent approach to CLD development across sub-system teams, internal guidance was developed by A.S., K.P. and M.W.

Kumu software (www.kumu.io) was used to represent CLDs visually. Factors (CLD ‘nodes’) and connections between nodes denoted hypothesized causal relationships. Feedback loops between nodes were identified using the automatic function in Kumu and interpreted by the sub-system team.

The first iteration of each CLD was presented in an internal workshop involving the wider Mandala research team. CLDs were reviewed and relationships between each of the sub-system CLDs were critically reviewed.

The second iteration of each sub-system CLD was developed through direct engagement with stakeholders. Approaches to stakeholder collaboration were tailored to sub-system stakeholder groups, acknowledging the barriers to and facilitators of engagement specific to each group. Engagement activities included stakeholder workshops using group model building techniques, one-on-one interviews and informal conversations with stakeholders [24]. The objectives of this collaboration were to refine the CLD in terms of accuracy and specificity for Birmingham and achieve consensus among stakeholders on how their part of the food system drives consumption of a healthy and sustainable diet.

#### (iv) Phase 2a: identifying intervention ideas

The generation of intervention ideas started with an analysis of the five sub-system CLDs to identify leverage points. A ‘leverage point’ was understood as ‘places in the system where a small change could lead to a larger shift in [system] behaviour’ [25, p. 145], through subsequent ‘knock-on effects’ or capitalization of system dynamics. Leverage points exist at different levels of system properties, with different levels of transformative potential [26]. According to Abson *et al.* [27], the least transformative leverage points lie in *system parameters* (material stocks and flows, actors and observable elements; e.g. presentation of meal options at point-of-sale) and *feedback* (e.g. procurement practices). The most transformative leverage points lie in *system design* (structure and rules; e.g. funding models), and *intent* (goals and paradigm; e.g. economic profit). It is more difficult to intervene on more transformative leverage points as intended change is less tangible, may face greater resistance owing to established interests or preferences and often requires coordinated efforts by multiple actors. However, change at this level can achieve transformation by removing pressure in the system to revert to original functioning [26,28,29].

There is currently no standardized, widely accepted method to qualitatively identify leverage points. Using sub-system CLDs as a tool for leverage point identification, the sub-system teams first met in an internal workshop, and then sub-system teams and stakeholders met either in interviews or in stakeholder workshops. Workshop and interview participants were introduced to the hierarchy of leverage points and asked to draw on their deep knowledge of specific parts of the system. To identify leverage points at the level of system feedback, feedback loops were analysed in terms of perceived dominance in contributing to system outcomes. Feedback loops were considered in terms of reinforcing loops—presenting opportunities to disrupt or strengthen existing momentum towards growth or decline, and balancing loops—presenting opportunities to disrupt stabilization processes that allow the system to suppress the effects of interventions elsewhere and self-organize to its original functioning [25]. For example, in the workshop for the community food sector, three parts of the CLD containing feedback loops were discussed in groups; these feedback loops were labelled: ‘ability to offer healthy/sustainable food in the sector’, ‘ability for individuals to access/use the sector’ and ‘consumption of food offer from sector’. CLD nodes perceived as structurally important owing to their perceived influence over other parts of the system (e.g. via information sharing) were assessed to identify leverage points in the design of the system (i.e. structure and rules). For example, in the institutional catering CLD, the ‘menu’ node was connected to many other nodes; these connections and this node were assessed as a potential leverage point.

Amenability to modification was assessed for candidate leverage point by considering whether there were actors in Birmingham who could (individually or collectively) steward action and whether there would likely be resistance to action. Finally, workshop and interview participants considered the system repercussions of acting on leverage points by following interconnected feedback loops or causal chains to forecast potential impact or suppression. Despite a focus on leverage points at the level of system feedback and design, leverage points at the level of system parameters and intent could also be discussed by participants and were recorded.

Interventions were considered as any actions (policies, activities, processes) that are implemented over a specified time period with the aim of achieving an effect on the specified outcome(s). Within a complex systems approach, such interventions are conceptualized as a designed *event* in a system, intended to disrupt patterns of system behaviour [18]. Consequently, interventions were firstly considered in terms of their function (i.e. their mechanism of action or *how* they will disrupt the system to produce a specified effect), rather than the form of the intervention (i.e. *what* will be implemented).

Solution scanning methods were adapted to gather additional intervention ideas from a rapid scan of 27 food system databases, food system reports and other sources [30]. To obtain ideas specific to Birmingham, suggestions from the public consultation on the Birmingham City Council’s Food System Strategy were included if they adequately targeted leverage points [10]. Finally, any interventions or activities that were already planned by stakeholders, and were understood to target identified leverage points, were considered at this or the following intervention design phase. For example, a stakeholder shared news of recent charitable funding to support a ‘healthy takeaway’; this activity was assessed in relation to the out-of-home retailing sub-system.

#### (v) Phase 2b: prioritizing interventions

A bespoke set of criteria was developed to assess intervention ideas against their hypothesized power to effect change (e.g. ‘action on system leverage points’), the evidence (e.g. ‘based on existing theory or theory developed in CLD-development'), the feasibility of implementation by collaborating stakeholders (e.g. ‘deliverability’; ‘cost of implementation’) and coherence with existing policy and other Mandala interventions (e.g. ‘alignment with existing interventions in Birmingham’)^1^ . First, the sub-system research team used a selection of the criteria to consider a long-list of intervention ideas for each sub-system. Ideas scoring poorly against criteria were omitted from a subsequent intermediate-list which was shared with stakeholders for supplementation and feedback on feasibility and need. Further omission of ideas deemed infeasible, unneeded or relatively less effective, cost-effective or evaluable resulted in a short-list of ideas. Short-listed ideas were prioritized through discussion with stakeholders who might deliver the interventions and in an internal ‘prioritization workshop’ involving the wider research team to consider synergies, system coverage and coherence across interventions. As an example, in the institutional catering sub-system: a long-list of 34 intervention ideas was reduced to 17 ideas in an intermediate-list, which was reduced to a short-list of ten ideas, of which four were of most interest to stakeholders and presented at the internal prioritization workshop; finally, two ideas were selected for the intervention design phase. This prioritization process resulted in a package of interventions across Mandala (table 2).

**Table 2.** List of specific interventions for Mandala Consortium explored in detail with stakeholders. CLD, causal loop diagram.

sub-system	intervention setting	intervention description	example challenges and opportunities for collaborative interdisciplinary systems approach to intervention design
out-of-home food retailing	an alternative, healthy and sustainable takeaway outlet	social business model utilizing surplus food—a healthier and more sustainable takeaway in a socio-economically deprived neighbourhood	limited opportunity for *collaboration* on intervention design as funding for initial phase of implementation obtained prior to Mandala involvement; anticipated use of data routinely collected by delivery stakeholders restricted opportunities for in-depth environmental analysis, limiting opportunities for *interdisciplinary* working; ability to act on leverage points in both out-of-home retailing and community food sector sub-*systems*
grocery retailing	convenience stores	promoting healthier and more sustainable product swaps for approximately 100 items in a sample of convenience stores served by one wholesaler in mainly socioeconomically deprived areas	anticipated that *collaboration* with stakeholders would be challenged by different prioritization of health, environmental and equity goals; intervention design activities performed with one expert practitioner who acted as a broker to end-users (i.e. convenience store owners, wholesalers); lack of funding for implementation stymied opportunities to design an intervention targeting health, environmental and economic outcomes (i.e. increased coverage of identified *system* leverage points), preventing anticipated *interdisciplinary* work
grocery retailing	a leading supermarket chain	enhancement of the national Healthy Start Voucher offer for food-insecure households with children	significant challenges engaging high-level decision-makers in *collaboration*; stakeholders responsible for health, social and environment goals could only act with agreement from more senior decision-makers, highlighting the importance of power dynamics in the wider food system
grocery retailing	home ordering from a recipe box company	working with one recipe box company to evaluate whether recipe boxes represent a scalable and widely acceptable way to promote healthier and more sustainable diets in households	close *collaboration* with stakeholders in setting-up a trial, facilitated by strong alignment of primary goals around healthy and sustainable grocery retail; *collaborative* intervention design activities focused on the offer (i.e. discounting structure) made to households; access to high-quality, diverse data routinely collected by delivery stakeholders facilitated *interdisciplinary* working
institutional catering	an NHS hospital	shifting institutional catering towards more plant-based food menus	early involvement of NHS stakeholders in stakeholder workshop to develop institutional catering CLD enabled process of *collaborative* design (i.e. shared decision-making) from the outset; design of intervention and implementation informed by development of diagnostic tools to estimate environmental impacts of menu design and meetings to share best practice from other settings; environmental, economic, public health, statistical and other methodological expertise required, emphasizing role of translational process facilitator for effective *interdisciplinary* working
institutional catering	primary schools	affordable school meal options—a cost–benefit analysis of alternatives to universal free primary school meals, including subsidized meals and targeted free school meals	*collaboration* processes focused on structured appraisal of policy options, allowing direct attribution to stakeholder input; engagement of stakeholders from regional/local government, academia and school leadership
community food sector	city wholesale market	developing and implementing a surplus food hub and network to supply community food organizations tackling food insecurity, based in the city wholesale produce market	alignment of goals with primary stakeholders facilitated authentic consultation, but fast implementation of intervention upon obtaining funding limited opportunities for more in-depth *collaboration* on design; setting of intervention in wholesale market demanded integration of insights from supply chains and wholesaling sub-*system***,** supporting *interdisciplinary* working

#### (vi) Phase 2c: designing interventions with stakeholders

Prioritized intervention ideas had thus far been considered in terms of their function. Where the form of the intervention was not yet apparent (i.e. intervention ideas that were not already planned activities in Birmingham), collaborative workshops and conversations with potential delivery stakeholders were held to design the form of the intervention and understand the required resources for delivery and evaluation [31]. For example, a workshop and a series of conversations was held with representatives from a third-sector organization in Birmingham to design an intervention aiming to support a network of suppliers and recipients of surplus food. This focused on the function of increasing system efficiency and reducing waste, as well as supplying fresh foods to the community food sector and reducing demand on organizations’ facilities and resources. Design discussions concerned the processes of recruiting organizations into the network, technical support for the network, and the possible design of an intervention to support surplus food redistribution to the community food sector. Specifically, during an initial workshop, presentations were used to structure a discussion around four intervention options: (i) utilization of a new or existing app to facilitate the supply and collection of surplus food before collection across a dispersed network, (ii) creation of a central facility to store surplus food before collection, possibly with an attached kitchen, (iii) embedding surplus food redistribution guidance in Environmental Health inspections for new outlets, and (iv) creation of a physical hub for surplus food storage and collection, supported by collective food-buying by network members. Stakeholders deemed options (i) and (iii) to be less feasible or desirable. Options (ii) and (iv) were merged and ideas around the operational model, location, delivery partners, resourcing, timelines and funding of a hub were generated through discussion. The design of the evaluation formed a final discussion point. Following the workshop, the delivery stakeholders continued to develop plans for implementation and seek funding, and the research team developed plans for evaluation; online meetings were used to share developments. As the package of interventions included some that were already planned, the design process was implemented along a spectrum of collaboration: from close collaboration and shared decision-making (adopting the principles of co-design [31]) to informed engagement between researchers and stakeholders.

Collaborative design processes allowed us to conclude that some interventions were not feasible to deliver or evaluate within the timeframe or resources of Mandala, which forced us to reappraise plans. For example, following discussion with stakeholders from local government and a primary school, the extension of free or subsidized school meals was deemed too expensive and unfeasible for delivery and evaluation at scale. However, a stakeholder workshop involving stakeholders from local and regional government, academia and school leadership enabled the development of a cost–benefit modelling study of four policy options as alternatives to universal free school meals, generating research with policy relevance where evaluative research was not possible [32].

Sub-system CLDs were pruned to focus on the system functions directly relevant to the intervention. The pruned CLDs formed the basis of a theory-of-system-change for each intervention, articulating inputs, outputs, outcomes and feedback loops. These articulated the desired function of the intervention in terms of changing system functioning, as well as potential unintended consequences. System-based theories-of-change formed the basis for evaluation design in future phases.

## 4. Findings: reflections on the challenges and opportunities of Mandala’s collaborative, interdisciplinary systems approach to urban food system transformation

Our analysis of the challenges and opportunities presented by Mandala’s approach is organized with reference to Freeth & Caniglia’s challenge dimensions [13]. We highlight where these dimensions could be identified, and therefore do not apply all dimensions to every aspect of Mandala’s approach. Key reflections on the challenges and opportunities of the approach when applied to intervention design are presented for a selection of intervention ideas in table 2.

### (a) Interdisciplinary science

The initial phases of Mandala involved approximately 20 researchers and three engagement and policy professionals spanning ten disciplines, across ten institutions in England. Despite our ambition for interdisciplinarity, we did not achieve this in every aspect. Challenges in the epistemic, symbolic, spatial and social dimensions shaped the engagement of different disciplines and ultimately led to a mix of ‘integrative interdisciplinarity’ and ‘additive multidisciplinarity’ across the sub-systems [13].

Epistemic differences were expected and informed learning and discussion around what constitutes a healthy and sustainable food system. These differences were shaped by disciplinary knowledge and prior engagement with various sectors and stakeholder groups. Epistemic differences produced variations in how types of evidence were valued, practices for developing stakeholder relationships and working with research participants, and specialized terminology (e.g. ‘system map’) and methods (e.g. what constitutes a systematic review). The importance of power differentials in the symbolic dimension meant that disciplines with more representation in Mandala (e.g. public health) had arguably more power to shape the ways of working, and that more experienced team members were responsible for building time and space for needed clarifications (e.g. what was meant by a ‘systems map’). For example, written guidance to support the consistencies of and consensus around research processes was intended to enable open and critical discussion across disciplines during their drafting. However, as the writing of guidance was initiated by researchers from public health and social science disciplines, it could have served to emphasize an uneven distribution of power. As such, this symbolic differential influenced epistemic, social, spatial and temporal dimensions relating to interdisciplinary working. However, a pluralistic approach to data collection and synthesizing evidence generated meaningful contributions across the disciplines, permitting use of diverse methods, including qualitative interviews, rapid solution scanning, scoping and systematic reviewing, CLD development and modelling.

Practically, delegation and leadership of non-specialized tasks needed to be negotiated and managed across a large team, creating challenges with the symbolic dimension as the project conformed to a conventional project structure with a Principal Investigator and senior co-investigators. Mandala’s experience confirmed existing research [33,34] on the role(s) of researchers in transdisciplinary/collaborative interdisciplinary projects as multi-faceted, going beyond that of scientist to encompass change agent, process facilitator and project worker. This extension of the researcher role was in conflict with the traditional reward systems of disciplinary science. For example, dedicating substantial time to creating and iterating the research process left little time for generating traditional outputs in these early phases, which was especiallychallenging for earlier career researchers.

While not inherent to interdisciplinary science, the different geographical locations of team members presented challenges in the spatial and social dimensions by limiting opportunities to meet in person. The use of digital platforms enabled regular formal and informal meetings but did not always offer appropriate configurations for learning opportunities in a large group, as they required facilitated, often prescribed discussion, rather than more informal and serendipitous conversations. Ensuring there were ample opportunities for updates, collaboration, building consensus and sharing learning across different formats, from small online meetings to full-team, in-person workshops, helped to navigate challenges in the spatial and social dimensions.

Finally, consideration of the temporal dimension highlighted the potential impact of determining the required disciplinary representation at the grant application stage of Mandala, limiting the scope to be reactive and integrate new disciplines once the research began.

### (b) Systems thinking

Adopting a complex systems approach is clearly aligned with the conceptualization of the food system as a complex adaptive system. However, within systems thinking, there are differences between hard, soft and critical systems approaches, which, respectively, adopt positivist, interpretivist and critical scientific perspectives. In what is now termed a ‘hard’ approach, the system under study is conceived to *exist in the world*; a model of a system attempts to be objective and singular in describing ‘what is the case’ [35]. In a ‘soft’ approach, the system under study is the *constructed conceptualization of a real-world situation* which is informed by stakeholders’ different experiences of that situation; models of a system cannot be singular as they are reflective of the various insights selected to build them [35,36]. A ‘critical’ approach incorporates reflexivity and attempts to acknowledge how power differentials shape a constructed system, such as through the definition of the boundaries of the system under study and the inclusion and dominance of certain stakeholders in the development of the conceptualization [37,38]. Moreover, the application of complex systems approaches is more established in some disciplines than others. For Mandala, this presented some challenges in epistemic, symbolic and temporal dimensions.

Epistemologically, there was a need to establish what would constitute ‘good knowledge’ of the system and which methods would enable us to achieve this knowledge while being sufficiently rigorous yet flexible (acknowledging a need to tailor methods, owing to our collaborative approach). In the first two phases of Mandala, systems thinking was used as a tool to integrate stakeholder insights into one understanding of the food system. Although our overall approach more closely aligned with a soft systems approach, this was not explicitly set out at the outset of the research. This meant that some research team members may have adopted a more interpretative stance on what constitutes ‘good knowledge’ than might be typical in their discipline, where positivist or critical epistemologies might be more common [39]. Interdisciplinary working helped to reveal these differences. Beyond the conceptualization of the system itself, a systems approach also asked researchers to accept the conceptualization of an intervention as an event in a unique, context-dependent system, rather than a set of activities that might generate effects that would generalize to other contexts [18]; again, this conceptualization may have involved a shift in thinking.

With respect to our chosen methods, it was important to maintain confidence in a novel approach to examining, intervening in and evaluating change in the food system. This required the research team to commit to a process that might iterate over time and was itself a focus of the research (a later work package will consider whether the research *process* was effective in identifying *transformative* interventions). Furthermore, the research process required researchers—and their funders—to accept that the focus of the evaluative research was to emerge from the research, rather than be stipulated in advance, as is more usually the case in academic research. This presented challenges in both epistemic and temporal dimensions, in terms of project and personnel management. Building consistency into the process as it emerged was facilitated by agreeing within the team written guidance on working internally and with stakeholders to develop a CLD, use the CLD to identify leverage points, generate and prioritize intervention ideas, and develop an intervention theory-of-change using the sub-system CLD. Utilization of this guidance also needed to be facilitated to ensure consistency.

CLDs have been proposed as an effective ‘epistemic boundary object’ to enable stakeholders and researchers to create a shared visualization of the system, stimulating collaboration [13]. The wide boundaries of the system under study in Mandala necessitated a focus on a series of sub-system CLDs. It was neither practicable nor possible for each researcher and stakeholder to contribute or deeply engage with each sub-system CLD. For stakeholders, where engagement did happen, CLDs did appear to offer a useful shared visualization of the system, although in some cases the complexity of the system maps was deemed challenging. The initial development of CLDs by the research team will have meant that their shared perspective was centred in the CLD, requiring stakeholders to first engage with that perspective to contest or support it, rather than first bringing-in their own perspective. For example, in the stakeholder workshop for the community food sector, stakeholders identified missing nodes or connections in the CLD and queried the polarity and direction of connections or the wording of node labels, but no stakeholders suggested the system should be presented in a vastly different way. Consideration of the epistemic and symbolic dimensions identifies this as a challenge in managing the power dynamics between researchers and stakeholders. It is possible that using participatory group model-building techniques earlier in the development of CLDs would have allowed us to construct a more consensual representation of the system.

A critical systems approach acknowledges the influence of power in constructing and observing systems. Symbolic power relationships are likely to have informed which stakeholders were approached by Mandala and which felt able or willing to contribute to the research. This will have influenced our visualization of the urban food system in the CLDs, our interpretation of system functioning through qualitative identification of leverage points and the prioritization and design of interventions. Acknowledging an influence of these power dynamics will be important when assessing the effectiveness of our methodological process and could in future be partially circumvented by using multiple techniques to engage stakeholders (e.g. using social network analysis to systematically identify key actors; putting out open calls for collaboration rather than operating through existing networks).

Stakeholders were viewed as partners in designing the intervention and were responsible for intervention delivery. This balance of power limited the ability for the research team to design ‘responsive adaption’ into the intervention, whereby interventions adapt in response to reaction from the rest of the system (a likely consequence of any event in a self-organizing system). Furthermore, it might have meant that the pragmatics of intervention delivery were necessarily prioritized over potential for transformative system change.

### (c) Stakeholder collaboration

In considering the full breadth of the urban food system, it was necessary to engage and collaborate with diverse groups of stakeholders. Collaboration with stakeholders presented valuable opportunities to understand food system actors’ mental models of how positive and adverse outcomes can emerge from a complex adaptive system. It also increased the likelihood of developing acceptable interventions that, if effective, could be embedded beyond the life of the Mandala research programme. Moreover, collaboration facilitated agile adaptation of our research processes, through timely learning of the existence and implications of internal or external shocks to the system, such as, respectively, changes in key personnel in potential delivery partner organizations or recent economic downturn.

Such opportunities could be threatened by challenges experienced in epistemic, social and spatial and temporal dimensions, with these challenges varying across different types of stakeholder group (e.g. commercial versus third sector). These challenges meant that collaboration efforts across sub-system teams achieved varying levels of meaningful participation: from stakeholders being informed and consulted, to intervention and evaluation designs being stakeholder- or researcher-initiated with shared decision-making [40].

Within the epistemic dimension, it might be expected that the perceived value of the research and food system change differed according to sectoral and organizational goals [41]. By foregrounding certain outcomes, we could reflect stakeholders’ organizational goals and priorities and present collaboration as an opportunity to inform beneficial interventions. Specifically: environmental objectives appeared important to public, commercial and third-sector organizations preoccupied with addressing community advocacy around climate issues or meeting formal ‘Net Zero’ targets; equity objectives were perceived as particularly important to third-sector organizations preoccupied with alleviating growing levels of food insecurity; and economic viability objectives appeared to be of particular importance to public and commercial organizations facing immediate or long-term economic competition or uncertainty. While diet-related health outcomes were stated as being of interest to most stakeholder groups, the research term perceived that—with the exception of public organizations—they could be given lower priority than other objectives (including other health outcomes such as hunger), or could even be in conflict with other objectives (e.g. owing to commercial profit being driven by unhealthy foods [42]). Usually, however, the act of foregrounding certain outcomes did not prevent the presentation of improvements to other outcomes as a co-benefit (e.g. reducing ruminant meat provision could improve environmental *and* health outcomes). Finally, for other groups, collaboration itself could align with organizational goals around building city-wide networks.

The intervention prioritization process may have underscored epistemic differences between researchers and different stakeholder groups, as the bespoke selection criteria reflected the priorities and perspectives of the research team, who then directed the application of criteria.

Conventionally, stakeholders from the private sector may be more resistant to participatory methods—such as group model-building methods, which encourage participants to share system insights—owing to perceived risks to competitiveness [43]. Conversely, stakeholders in the public or third sector may be more familiar or comfortable with such exercises. We tailored our research methods to navigate these challenges in the social dimension, for example by offering face-to-face interviews to review CLDs and generate intervention ideas, as an alternative to participatory workshops. However, tailored methods may have limited deeper engagement by individual stakeholders (e.g. less time in interviews to elaborate on a systems approach and new ways of viewing the problem) and between stakeholders, impacting our ability to foster a shared understanding of the system within certain stakeholder groups.

Finally, in the temporal dimension, time-scarce stakeholders presented a challenge, demanding sensitive scheduling of collaborative activities to recognize different working patterns (e.g. busy periods during meal service for institutional caterers; seasonal demands on the commercial and community food sectors). The sequencing and timing of collaborative activities throughout the research was also challenging. It was difficult to maintain communication and commitment owing to a lengthy period of intervention identification and prioritization (necessitated by the adoption of a complex systems approach) and a high degree of ambiguity around intervention delivery until final prioritization and intervention design. In addition, as interventions were to be delivered by stakeholders rather than the research team, securing timely commitments to hypothetical intervention delivery was complicated on *both* sides (researcher and stakeholder) and risked disrupting relationships with stakeholders. To overcome these challenges, it was useful to explicitly communicate the research process and reasons for ambiguity. Incorporating both formal and informal engagement activities was found to help maintain relationships and provide timely project developments and timelines to stakeholders. Such activities might traditionally be seen as outside the scope of researcher duties, and therefore needed to be explicitly acknowledged when considering project resources [19].

## 3. Discussion

Mandala iteratively developed a research process combining interdisciplinary science, systems thinking and stakeholder collaboration to identify evaluable interventions in the urban food system aiming to support healthy and sustainable diets. Freeth & Caniglia’s framework helped us to systematically expose the challenges and opportunities of moving towards a collaborative interdisciplinary systems approach in this endeavour [13]. Challenges were overcome to varying degrees through: the tailoring of our research process to suit different stakeholder groups; the use of written internal guidance to support stakeholder collaboration and systems thinking; a planned pluralistic approach to data collection and evidence synthesis; and a pluralistic approach to evidence generation to include modelling as well as evaluation.

The challenges pertaining to stakeholder collaboration varied across stakeholder groups; notably, the perceived value of the research (and food system change) and the acceptability of participatory methods differed according to sectoral goals and conventional practices. Challenges and opportunities arising from the adoption of a complex systems approach lay in epistemic, symbolic and temporal dimensions, partly owing to disciplinary leanings towards hard, soft and critical systems approaches. Project and personnel management was also challenged by an emergent research process necessitated by a systems approach. Finally, interdisciplinary science evoked opportunities and challenges in all dimensions except the temporal and provided fertile ground for interdisciplinary learning and a pluralistic approach to data collection, integration and interpretation.

In integrating three methodological principles, Mandala aimed to operationalize a collaborative interdisciplinary systems approach that allowed us to: derive conceptual models from multiple perspectives; integrate multiple goals and research questions when designing interventions and evaluations; ensure transparent and frequent communication to maintain momentum and relationships; and respond agilely to change in the systems in which we work (figure 1). Our findings suggest that we were able to achieve many of these aspects, but overcoming further challenges will enable us to do this more effectively. Future evaluative evidence from Mandala will provide insights into whether our realization of a collaborative interdisciplinary systems approach allowed us to identify interventions with transformative potential. We suggest that other research adopting a similar approach could: allocate time and space to elucidating epistemic differences in interdisciplinary teams by building definitional foundations and engaging with epistemic boundary objects; use multiple techniques to recruit stakeholders in order to reduce the influence of power differentials between researcher and stakeholder; allocate sufficient resources to engagement activities to build and maintain stakeholder relationships throughout the research process; and explicitly acknowledge and reward the extension of the researcher role to encompass change agent or process facilitator [33,34].

### (a) Strengths and limitations

We adopted Freeth & Caniglia’s framework to systematically appraise our methodological approach [13]. This is thought to be valuable in addressing complex problems but has relatively few worked examples in the literature. Our appraisal is based on our own reflections as members of the research team. While this is a limitation in terms of subjectivity; it also affords unique insights owing to our deep embeddedness in the research and experiential knowledge, which would not be afforded to an external observer. To provide a complete appraisal of our interdisciplinary systems approach, future research will assess whether our package of interventions and evaluations affected systems change and whether we could maintain application of the three methodological approaches through the final phases of Mandala.

### (b) What this study adds

Our findings suggest there are benefits to moving closer towards a collaborative interdisciplinary systems approach, particularly for research addressing problems that, owing to their complex and complicated nature, have defied traditional approaches. However, there are challenges in doing so. Freeth & Caniglia consider research collaborations across epistemic, social, symbolic, spatial and temporal dimensions [13]. These dimensions can be used across different research programmes and projects to identify and address challenges going forwards and enable innovation and shared learning.

As noted above, some dimensions were more readily identifiable than others in the Mandala case study. It was also sometimes difficult to distinguish the dimensions from one another—for example, there appear to be significant overlaps between social (different ways of being together, and relations with peers and competitors) and symbolic (about how power dynamics operate between collaborators, and shape values, norms and expectations) dimensions. In extending the Freeth & Caniglia framework beyond researcher–researcher collaboration to researcher–stakeholder collaboration, we believe we have enhanced its utility [13].

### (c) Implications

To ensure the adoption of a collaborative interdisciplinary systems approach is multiplicative rather than additive, concerted efforts are needed to embed interdisciplinary and collaborative working and integrate different conceptual perspectives, theories and methods [13]. This may involve a preparedness to disrupt conventional research roles, timelines, processes and allocation of resources, which may be drawn from a dominant field or discipline. Moreover, the challenges identified in this paper may only be overcome with the support of different research funding processes, such as greater flexibility in budgetary management so that resources can be deployed when needed, rather than according to a prespecified profile.

### (d) Unanswered questions and future research

To provide a complete appraisal of our collaborative interdisciplinary systems approach, planned research in Mandala will assess: the prioritization process of intervention ideas using a framework of systems-level change (e.g. intervention-level framework) to reveal whether more transformative interventions were (de)selected; whether our package of interventions and evaluations resulted in systems change; and whether we could maintain application of the three methodological approaches through the final phases of Mandala. Finally, repeat surveys and semi-structured interviews will be used to explore the research team and stakeholders’ perspectives on the research process and our collaborative interdisciplinary systems approach. Insights will allow us to build upon our experiential knowledge of what was good practice and what continued to present challenges.

Our analysis of the symbolic dimension in this paper highlights the potential value of explicitly adopting a critical systems perspective from the outset of the research process. Future research adopting such an approach could pre-empt and negotiate the impact of unequal power dimensions on research aiming to understand and act on complex systems, and therefore include a broader range of stakeholders (for example citizens) where appropriate and support interdisciplinary working.

Finally, future use of the Freeth & Caniglia framework to explore researcher–stakeholder collaboration should interrogate whether some dimensions are more applicable to researcher versus stakeholder collaboration.

## 4. Conclusion

Society increasingly faces intractable and systemic problems that demand solutions drawing on knowledge and expertise from multiple disciplines and sectors. The urgency to address such problems intensifies as the societal, human, environmental and economic costs mount. New methodological approaches for research and innovation are required to unpack these problems and develop feasible and evaluable interventions that have the power to catalyse scalable and sustainable change. A collaborative interdisciplinary systems approach may represent such an approach. Confronting the challenges of this new methodological approach should be a shared endeavour that will be expedited by transparent reporting of processes and methods.

## Data Availability

Supplementary material is available online [44].

## Footnotes

^1^
The development and detailed application of the criteria are the subject of a separate publication.
